# Alterations of miRNAs and Their Potential Roles in Arsenite-Induced Transformation of Human Bronchial Epithelial Cells

**DOI:** 10.3390/genes8100254

**Published:** 2017-10-03

**Authors:** Shiyan Gu, Donglei Sun, Xinyang Li, Zunzhen Zhang

**Affiliations:** Department of Environmental and Occupational Health, West China School of Public Health, Sichuan University, Chengdu 610041, China; ygsy727@163.com (S.G.); sundonglei2016@yahoo.com (D.S.); lixinyang221bc@163.com (X.L.)

**Keywords:** arsenite, transformation, microRNAs, Gene Ontolog, KEGG pathway analyses

## Abstract

The alterations of micro RNAs (miRNAs) and their potential roles in arsenite-induced tumorigenesis are still poorly understood. In this study, miRNA Array was used to detect the expression level of miRNAs in human bronchial epithelial (HBE) cells that were transformed by 2.5 μM arsenite for 13 weeks. These cells exhibited a neoplastic phenotype manifested by increased levels of cellular proliferation and migration and clone formation. Subsequently, 191 dysregulated miRNAs were identified to be associated with arsenite-induced transformation by miRNA Array. Among them, six miRNAs were validated by their expression levels with quantitative real-time polymerase chain reaction (qPCR), and 17 miRNAs were further explored via their target genes as well as regulatory network. Three databases, TargetMiner, miRDB, and TarBase, were used to predict the target genes of the 17 miRNAs, and a total of 954 common genes were sorted. Results of Gene Ontology (GO) analyses showed that the 954 genes were involved in diverse terms of GO categories, such as positive regulation of macroautophagy, epithelial cell maturation, and synaptic vesicle clustering. Moreover, results of Kyoto Encyclopedia of Genes and Genomes (KEGG) pathway analyses demonstrated that most of these target genes were enriched in various cancer-related pathways, including non-small cell lung cancer, Wnt signaling pathway, cell cycle, and p53 signaling pathway. The miRNA-gene regulatory network, which was constructed by cytoscape software with miRNAs and their target genes, showed that miR-15b-5p, miR-106b-5p, and miR-320d were the core hubs. Collectively, our results provide new insights into miRNA-mediated mechanisms underlying arsenite-induced transformation, although more experimental verification is still needed to prove these predictions.

## 1. Introduction

Arsenite, a major form of inorganic arsenite found in drinking water, food, and air, poses a threat to more than 19.6 million people in China [1]. Epidemiological studies have demonstrated that chronic exposure to low concentrations of arsenite can significantly increase the risk of a variety of cancer types, such as skin, bladder, and lung cancers [2], and the mechanisms underlying these tumors have attracted widespread attentions. Cell malignant transformation is an optimal model to determine the mechanism of carcinogen-induced tumorigenesis due to the fact that the process of transformation is closely related to the process of tumor occurrence and development. More importantly, there are many similarities in neoplastic phenotype of tumor cells and arsenite-transformed cells, including typical cellular morphological changes, powerful migration and invasion potential, anchorage-independent growth, or/and epithelial-mesenchymal transition [3,4,5]. Therefore, the transformation model constructed by continually exposure to low concentrations of arsenite has been employed to extensively investigate arsenite carcinogenicity. A growing body of studies have evidenced that low levels of arsenite can transform several types of cells, such as human osteogenic sarcoma cells [6], human keratinocytes cells [7], human bronchial epithelial cells [8,9], and human prostate epithelial and stem cells [10]. With the help of these transformation models, oxidative stress [6], DNA damage, cellular apoptosis and autophagy [7,9], and aberrant epigenetic regulation [10] were proposed to be associated with the carcinogenicity of arsenite. However, the specific molecular mechanisms underlying arsenite affecting these biological events are still not fully understood.

Micro RNAs (miRNAs) are endogenous, single-stranded, and non-coding RNA (ncRNA) molecules with a length of 18–25 nucleotides [11]. Despite the relatively short time since the first miRNA discovery in *Caenorhabditis elegans* [12], 2588 mature miRNAs (1881 miRNA precursors) of *Homo sapiens* have been recorded in the miRBase database [13] so far. Numerous studies suggest that a single miRNA is able to control the expression of hundreds of protein coding genes and modulates a wide spectrum of biological functions, such as cell development, differentiation, apoptosis, DNA repair, and cell adhesion and motility, all of which are fundamental to tumorigenesis [11,14,15]. Importantly, it is now widely recognized that miRNAs are dysregulated in a variety of human cancers and act as key molecules to regulate the cancerous occurrence and progression [15,16]. 

Recently, the critical roles of miRNAs in arsenite-induced carcinogenesis are increasingly emphasized. miR-21, one of the most common miRNAs in tumorigenesis [17], is related to the key biological events in arsenite-induced transformation, including generation of reactive oxygen species [3], development of epithelial-mesenchymal transition and invasive potential [18], and activation of STAT3 signaling pathway [4]. Silencing of let-7c was closely associated with the arsenite-induced neoplastic transformation of human keratinocytes [19]. Moreover, induction of miR-190 can accelerate proliferation of human bronchial epithelial cells in the process of arsenite-induced carcinogenesis [20]. Furthermore, inhibition of miR-191 expression attenuated the epithelial-mesenchymal transition and decreased cellular migratory capacity as well as neoplastic properties in arsenite-transformed cells [21]. These studies together suggested that miRNAs play critical roles in the process of arsenite-induced transformation. However, more than 2000 miRNAs have been identified at present, and these miRNAs can regulate thousands of protein coding genes that mainly contribute to various cellular functions and signaling pathways [11,15,16]. Such studies of single miRNA-mediated one signaling pathway are insufficient to explain the complex molecular mechanisms of miRNAs in arsenite carcinogenesis. Therefore, the roles of miRNAs in tumorigenesis of arsenite need to be further studied.

In this study, the difference in expression profile of miRNAs between parent cells and arsenite-transformed cells was identified by miRNA Array. Our results showed that 191 dysregulated miRNAs are associated with the neoplastic transformation induced by arsenite. Three databases, TargetMiner, miRDB, and TarBase, were subsequently applied to predict the target genes of 17 dysregulated miRNAs, and a total of 954 target genes were sorted. Results from Gene Ontology (GO) and Kyoto Encyclopedia of Genes and Genomes (KEGG) pathway analyses of the 954 genes revealed that some specific molecular events and signaling pathways with tumor characteristics were related to arsenite-induced transformation. The interactions of miRNAs and their target genes were shown in miRNA-gene regulatory network, in which miR-15b-5p, miR-106b-5p, and miR-320d were the core hubs. Our results demonstrated that a set of miRNAs was possibly involved in the neoplastic transformation induced by arsenite and provided a new insight into understanding the key mechanisms of miRNAs in carcinogenicity of arsenite.

## 2. Materials and Methods

### 2.1. Cell Culture

Human bronchial epithelial (HBE) cell line was generously provided by the Stem Cells and Tissue Engineering Laboratory, State Key Laboratory of Biotherapy, Sichuan University, China. The cells were cultured in high glucose Dulbecco’s modified Eagle’s medium (DMEM, Life Technologies/Gibco, Grand Island, NY, USA) supplemented with 10% (*v*/*v*) fetal bovine serum, 100 units/mL penicillin, and 100 μg/mL streptomycin. Cultures were maintained at 37 °C in a humidified atmosphere containing 5% CO_2_ and 95% air. 

### 2.2. Establishment of the Arsenite-Induced Neoplastic Transformation Cells

In China, the current maximum contaminant level for arsenite in the drinking water is 50 μg/L (about 0.4 μM). However, high levels of arsenite (ranging from about 0.5 to 16 μM) in the ground water have been found in 19 provinces in China [22,23]. In addition, it has been reported that exposure to 1 μM arsenite for 15 weeks can transform human bronchial epithelial cells [21]. Therefore, in this study, in order to adequately simulate the environmental exposure concentration and shorten the time of constructing a neoplastic transformation cell model, we used a 2.5 μM concentration of arsenite for transforming the HBE cells. Briefly, HBE cells were continuously maintained in the medium containing 0 or 2.5 μM sodium arsenite (NaAsO_2_, purity: 99.0%, Sigma, St. Louis, MO, USA) for 24 h per passage. This process was continued for 40 passages (about 13 weeks), and these NaAsO_2_ treatment cells were termed as HBE-T cells. The growth characteristics and neoplastic phenotype of cells were observed under the microscope during the process of NaAsO_2_ exposure.

### 2.3. MTT Assay

The cellular proliferation capacity of HBE and HBE-T cells was detected by MTT [3-(4,5-dimethylthiazol-2-yl)-2,5-diphenyl-tetrazolium bromide] assay. In brief, HBE and HBE-T cells were seeded in 96-well plates at 2 × 10^3^ cells/well overnight, and cultured for additional 0 h, 24 h, 48 h, 72 h, and 96 h, thus allowing the cells to grow. At the end of each designated time, cells were then incubated with 100 µL of 0.5 mg/mL MTT at 37 °C for 4 h in the dark. Subsequently, 100 µL of dimethylsulfoxide was added to dissolve formazan crystals. Absorbance at 570 nm (A_570_) was measured with a micro-plate reader (Multiskan^TM^GO, Thermo Fisher Scientific, Inc., Waltham, MA, USA). The cell proliferation rate was calculated using the formula: cell proliferation rate (%) = T_n_A_570_/T_0_A_570_ × 100%. In this formula, T_n_A_570_ was the absorbance at 570 nm of cells incubation for 24 h, 48 h, 72 h, and 96 h. T_0_A_570_ was the absorbance at 570 nm of cells cultured overnight.

### 2.4. Plate Colony Formation Assay

HBE and HBE-T cells were seeded in 12-well plates at 1000 cells/well and cultured for an additional 14 days, allowing the colonies to form. Cells were then washed with PBS, fixed in methanol, and stained with 10% (*w*/*v*) Giemsa. Subsequently, colonies were viewed and counted under a microscope (Eclipse TiU, Nikon Corp., Tokyo, Japan), and their pictures were taken with a digital camera (Canon, Daejeon, Tokyo, Japan). The percentage of colony formation for each group was calculated with the equation: percentage of colony formation (%) = the number of colony/1000 × 100. 

### 2.5. Soft Agar Clone Formation Assay

The soft agar plates were prepared in 96-well plates with under-layers of 1.0% agar in DMEM supplemented with 10% FBS. To test the soft-agar clone growth capacity, HBE and HBE-T cells were seeded in the prepared 96-well plates at 500 cells/well with 100 µL of 0.5% agar in DMEM supplemented with 10% FBS. These cells were then incubated at 37 °C in a humidified 5% CO_2_ atmosphere, and the cultures were fed every three days. After two weeks, clones were examined microscopically and photographed with a microscope (Eclipse TiU, Nikon). The percentage of clone formation for each group was calculated with the equation: percentage of clone formation (%) = the number of clone/500 × 100. 

### 2.6. Wound-Healing Cell Migration Assay

A wound healing assay was performed to detect the migratory potential of HBE and HBE-T cells. In brief, cells were seeded into 6-well plates and cultured to form confluent monolayers. Cell monolayers were then scratched in a straight line to create a “wound” with a 200 μL pipet tip and washed twice with PBS. The width was photographed immediately after scratching (0 h) and after undergoing 48 h incubation by microscopy. The experiments were independently repeated three times. Cell migration distance was analyzed and calculated by Image-Pro Plus software (Media Cybernetics, Inc., Rockville, MD, USA). Fold change in cell migration = the migration distance of HBE-T cells/the migration distance of HBE cells. The migration distance of cells = the width of wound at 0 h—the width of wound at 48 h.

### 2.7. RNA Isolation

Total RNA including miRNAs were extracted from the HBE and HBE-T cells with a miRNeasy Mini kit (QIAGEN Gaithersburg, Inc., Gaithersburg, MD, USA) according to the manufacturer’s protocols. The quality of total RNA was quantified by absorbance at 280 nm, 260 nm and 230 nm using a NanoDrop 2000 spectrophotometer (ThermoFisher Scientific, Rochester, NY, USA). For spectrophotometer, the A_260_/A_280_ ratio between 1.8 and 2.1 are acceptable and the A_260_/A_230_ ratio should be more than 1.8. RNA integrity and gDNA contamination were further analyzed by denaturing agarose gel electrophoresis. 

### 2.8. miRNA Array and Data Analysis

To identify the changes of miRNAs expression profile in carcinogenesis induced by arsenite, miRCURY^TM^ Array (miRNA Array, Exiqon, Woburn, MA, USA) was used to detect the miRNAs between HBE and HBE-T cells. Briefly, RNA samples, mixtures of three independent experimental RNA isolation, were labeled with the Exiqon miRCURY Hy3/Hy5 power labeling kit and hybridized on the miRCURY^TM^ Array station. An Axon GenePix 4000B microarray scanner was used to scanning. GenePix pro version 6.0 was used to read image raw intensity. The intensity of green signal was calculated after background subtraction, and four replicated spots of each probe on the same slide were averaged. The median normalization method was used to acquire normalized data (foreground minus background divided by median). The median was the 50th percentile of miRNA intensity and was >50 in all samples after background correction. The threshold value, a fold change >2.0 was used to define upregulation or downregulation of miRNAs. Hierarchical clustering was performed to show the distinguishable miRNA expression patterns among samples. 

### 2.9. Fluorescence Quantitative Real-Time Polymerase Chain Reaction

We determined the level of six miRNAs (miR-33b-5p, miR-15b-5p, miR-192-5p, miR-141-3p, miR-200b-3p, and miR-106b-5p) to validate the reliability of the miRNA Array detection. The main reasons for selecting these miRNAs are as follows. First, these six miRNAs were changed markedly between normal and arsenite-transformed cells from our miRNA Array data (Table S1). Second, these miRNAs were more extensively studied and closely associated with lung cancer occurrence and progression [24,25,26,27,28,29]. The level of miRNAs was measured using fluorescence quantitative real-time polymerase chain reaction (qPCR) assay. Briefly, complementary DNA was synthesized from total RNA (<200 nt) via a Tiangen Reverse Transcriptase Kit (Tiangen Biotech, Beijing Co., Ltd, Beijing, China). Then, an equal amount of each complementary DNA sample was subjected to fluorescence quantitative real-time polymerase chain reaction (qPCR) analysis on Bio-Rad using a miRcute miRNA qPCR Detection Kit (Tiangen Biotech, Beijing Co., Ltd). The endogenous RNA species, U6, was used to normalize the miRNA expression levels. All the forward primers of miRNAs and U6 were purchased from Tiangen Biotech, and the reverse primer was provided in the miRcute miRNA qPCR Detection Kit. The relative expression level of each miRNA was analyzed through the comparative threshold cycle method through the equation 2^−ΔΔCt^ (ΔCt = Ct_miRNA_ − Ct_U6_, ΔΔCt = ΔCt_HBE-T_ − ΔCt_HBE_). The experiments were independently repeated three times.

### 2.10. Bioinformatics Analysis

Among the 191 dysregulated miRNAs, seventeen miRNAs (downregulation miRNAs: miR-197-3p, miR-192-5p, miR-127-3p, miR-139-5p, miR-490-3p, miR-196b-5p, miR-125a-3p, miR-298, miR-542-3p, miR-15b-5p, miR-33b-5p; upregulation miRNAs: miR-200b-3p, miR-106b-5p, miR-574-5p, miR-320d, miR-200c-3p, miR-141-3p, Table S2) were selected for bioinformatics analysis. The selection of these 17 miRNAs mainly based on the principle of selecting 6 miRNAs for validation that mentioned above in Section 2.9, and those miRNAs involved in the transformation process induced by other carcinogen were taken into consideration as well. Target genes of these 17 miRNAs were searched in three public databases: TargetMiner [30], miRDB [31] and TarBase [32]. The numbers of predicted target genes were then presented in a Venn diagram [33], and the genes in the intersection of the three databases were chosen for GO and KEGG pathway analyses. GO categories and the pathways were classified by FunNet algorithm, and the corresponding results were evaluated with a unilateral Fisher exact test. False discovery rate (*FDR*) analysis was calculated to correct the *P* value. Enrich factor was calculated according to the following formula: enrich factor = (the number of different genes in a term/total number of differentially expressed genes)/(the total number of genes in the database term/total number of genes in the database). Enrich factors of GO categories and KEGG pathways in the top 30 were selected and presented in this study. Finally, the regulatory network of miRNAs and their target genes was constructed via Cytoscape software.

### 2.11. Detection of Cell Cycle Distribution

Cell cycle distribution was detected by flow cytometry using propidium (PI) staining. Briefly, 10^5^ HBE and HBE-T cells were collected, re-suspended and fixed in 70% (*v*/*v*) ethanol at 4 °C overnight. After incubating with PI staining solution (50 µg/mL PI, 20 µg/mL Ribnuclease A, 0.2% Triton X-100) for 30 min in the dark, cells were subsequently analyzed by using flow cytometer (Beckman Coulter, FC500, Brea, CA, USA), and 20,000 cells were measured for each sample. The percentage of cells in different cell cycle phase was analyzed using Multicycle for Windows (Phoenix Flow Systems, San Diego, CA, USA).

## 3. Results

### 3.1. Identification of Neoplastic Phenotype

**Figure 1:**
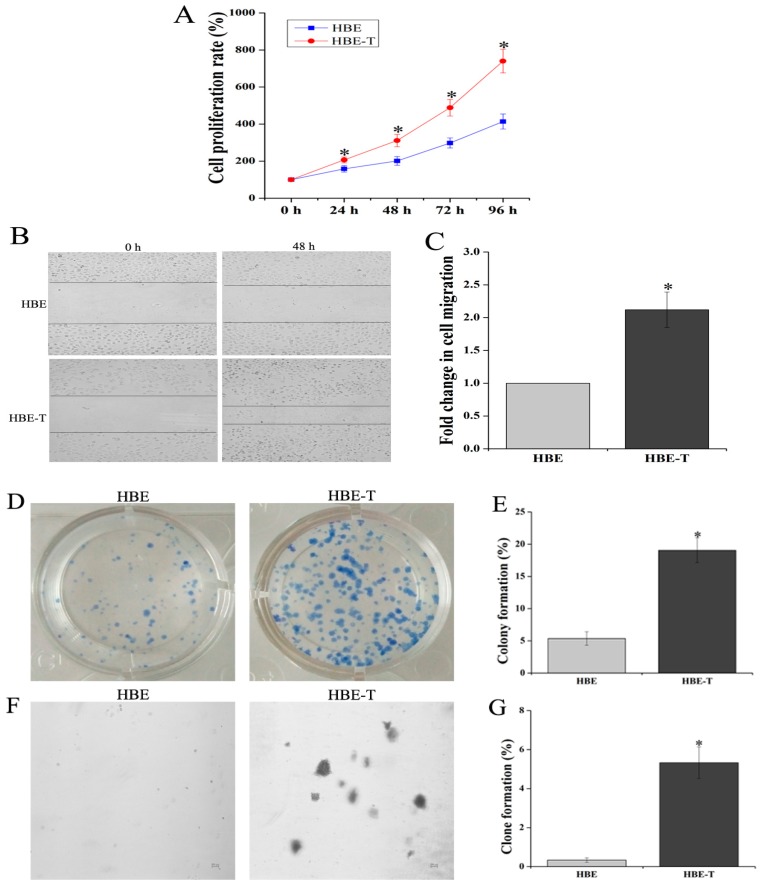
Identification of neoplastic phenotype of arsenite-transformed cells. Cells were treated with 2.5 μM arsenite for 40 passages (about 13 weeks), and the neoplastic phenotype of these cells was assessed comprehensively by MTT [3-(4,5-dimethylthiazol-2-yl)-2,5-diphenyl-tetrazolium bromide] assay, cell migration assay, and clone formation and colony formation assay. (**A**) Cell proliferation rates of human bronchial epithelial (HBE) and HBE-T cells; (**B**) Representative images of HBE and HBE-T cells in cell migration assay (40×); The fold change in cell migration was shown in (**C**); Panel (**D**,**F**) illustrate representative images of plate colony formation and soft agar clone formation assay; The rates of colony formation and clone formation were presented in (**E**,**G**). All the results were obtained from three independent experiments and are presented as mean ± standard deviation (SD). *p*-Values were calculated using independent samples *t* test, and * *p* < 0.05 is considered significant as compared with normal HBE cells.

MTT assay was performed to characterize the proliferation rate of HBE and HBE-T cells. Results demonstrated that the cell proliferation rates of HBE-T cells were higher than that of HBE cells after 48 h incubation (*p* < 0.05) (Figure 1A). To further test the migratory potential of HBE-T cells, cell migration assay was conducted. As shown in Figure 1B,C, HBE-T cells exhibited 2.11-fold higher migration distance than HBE cells. Moreover, since the potential of single cell growth is one of the most recognized indicator for identification cell neoplastic phenotype, plate colony formation and soft agar clone formation assay were both used to compare the potential of single cell proliferation between HBE and HBE-T cells. With the incubation of two weeks, HBE cells exhibited only 5.37% of colony formation and 0.33% of clone formation (Figure 1D,E), whereas HBE-T cells exhibited 19.07% and 5.33% (Figure 1F,G). These results together suggested that exposure to low concentration of arsenite for a long time could confer tumor characteristics to HBE cells.

### 3.2. The Identification of Aberrant miRNAs in Arsenite-Transformed Cells

In this study, we detected the miRNA expression profile in HBE and HBE-T cells using miRNA Array. Before measurement, the quality of RNA samples from HBE and HBE-T cells was tested by denaturing agarose gel electrophoresis and the results indicated that the RNA samples from HBE and HBE-T cells were in conformity with the requirements for following analysis (Figure S1A). A total of 2081 miRNAs were then detected in HBE and HBE-T cells and the data demonstrated that miRNA expression profile in HBE-T cells was obviously different from that in HBE cells (Supplemental Figure S1B). The calculated data further demonstrated that 191 miRNAs (128 miRNAs low expressed and 63 miRNAs high expressed, Table S1 and Supplemental Figure S1C) were aberrant in HBE-T cells when compared to HBE cells. 

### 3.3. The Results of qPCR Validation

**Figure 2:**
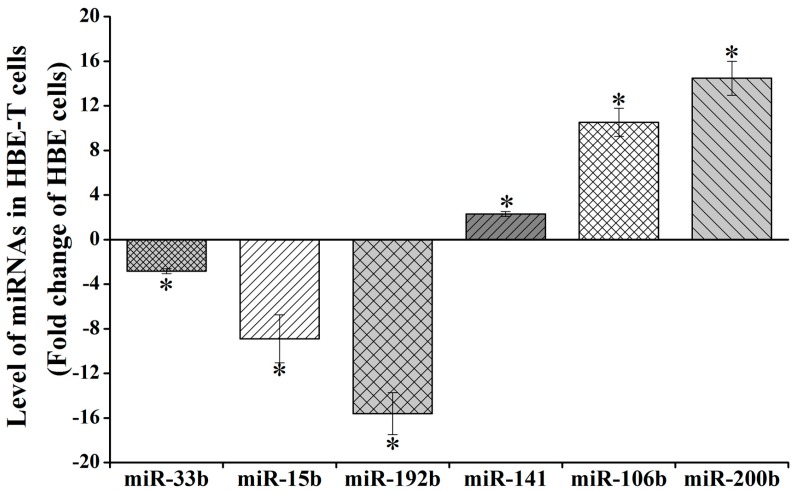
Validation for aberrant miRNA expression using qPCR assay. Total RNA including miRNAs were extracted from HBE and HBE-T cells and the miRNA level (miR-33b-5p, miR-15b-5p, miR-192-5p, miR-141-3p, miR-106b-5p, miR-200b-3p) was detected using qPCR assay. Level of miRNAs in HBE-T cells was normalized to corresponding miRNA level in HBE cells. Data were obtained from three independent experiments and quantitative results are presented as mean ± standard deviation (SD). *p*-Values were calculated using independent samples *t* test and * *p* < 0.05 is considered significant.

Three of downregulated miRNAs (miR-192b-5p, miR-15b-5p, and miR-33b-5p) and three upregulated miRNAs (miR-141-3p, miR-106b-5p, and miR-200b-3p) (Table S2) were selected for validating the reliability of analysis results from miRNA Array. As shown in Figure 2, the expression levels of miR-33b-5p, miR-15b-5p and miR-192b-5p in HBE-T cells were 0.36 (−2.82), 0.12 (−8.90), and 0.06 (−15.61) times of that in HBE cells. Also, miR-141-3p, miR-106b-5p, and miR-200b-3p expression levels of HBE-T cells were 2.29-, 10.51-, and 14.47-fold of that in HBE cells. Despite the fact that the values of these miRNAs are not exactly same with the values from miRNA Array analysis (Table S2), their changing trends were consistent. These results indicate that data from the miRNA Array analysis were reliable.

### 3.4. GO and KEGG Pathway Analysis

**Figure 3:**
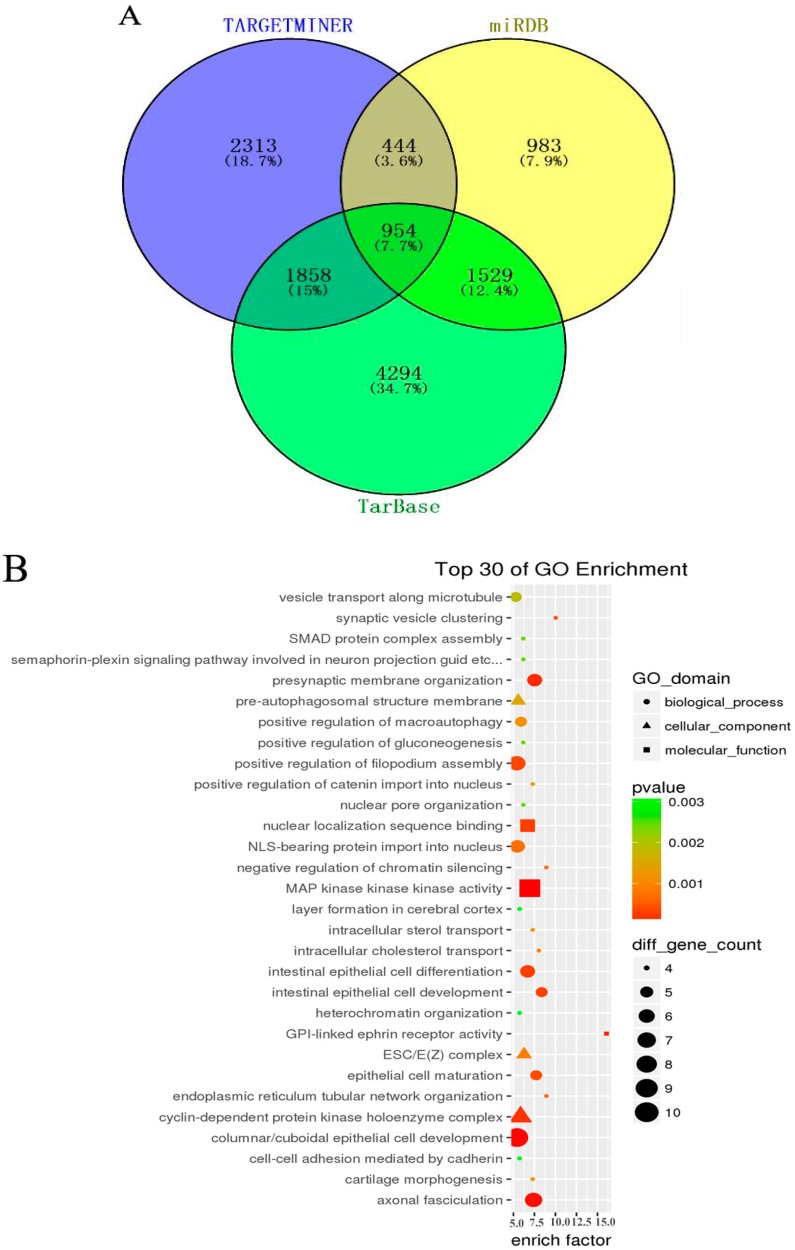

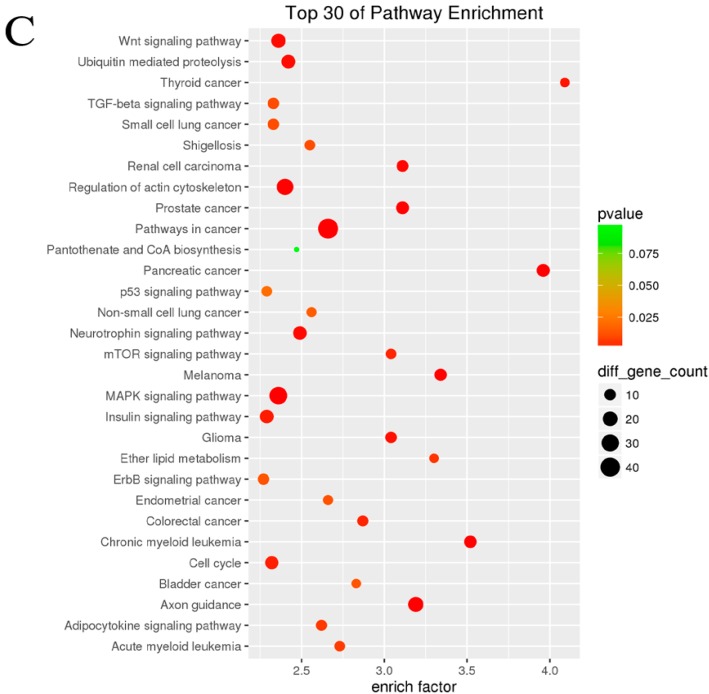
Gene Ontology (GO) and Kyoto Encyclopedia of Genes and Genomes (KEGG) pathway analysis for target genes of aberrant miRNAs. Three databases—TargetMiner, miRDB, and TarBase—were used to predict target genes of seventeen altered miRNAs (11 downregulated and six upregulated) between HBE and HBE-T cells. These predicted target genes were analyzed with a Venn diagram, and the genes in the intersection of three databases were used for GO and KEGG pathway analysis. (**A**) Venn diagram showing the common and unique target genes in TargetMiner, miRDB, and TarBase. The number and percentage in each section of Venn graph respectively represent the number of miRNA-gene pairs and the percentage of these genes account for of the total number of genes in three databases; (**B**) Top 30 of GO categories enrichment for the miRNA target genes in ontology of biological processes, cellular component and molecular function (*p* < 0.05 and *FDR* < 0.05); (**C**) KEGG pathways analysis was performed to investigate the miRNA target genes and the top 30 of pathways enrichment with *p* < 0.05 and *FDR* < 0.05 were selected and presented.

In order to further understand the role of aberrant miRNAs in physiological functions and biologic processes in arsenite-induced neoplastic transformation cells, 11 downregulated miRNAs (miR-197-3p, miR-192-5p, miR-127-3p, miR-139-5p, miR-490-3p, miR-196b-5p, miR-125a-3p, miR-298, miR-542-3p, miR-15b-5p, and miR-33b-5p) and six upregulated miRNAs (miR-200b-3p, miR-106b-5p, miR-574-5p, miR-320d, miR-200c-3p, and miR-141-3p) (Table S2) were selected, and their target genes were predicted with the TargetMiner, miRDB, and TarBase databases. There were 5569 miRNA-gene pairs in TargetMiner, 3910 miRNA-gene pairs in miRDB, and 8635 miRNA-gene pairs in TarBase. The numbers of these miRNA-gene pairs are presented in a Venn diagram (Figure 3A). The intersection genes of three databases (954 genes, accounted for 7.7% of all target genes in three databases) were identified as the accepted target genes of these 17 miRNAs. GO and KEGG pathway analyses were subsequently performed to investigate these target genes enriched in specific GO categories and pathways. As shown in Figure 3B, the top 30 of GO categories enrichment were selected and presented. GO biological process analysis showed that the target genes participated in negative regulation of chromatin silencing, epithelial cell maturation, endoplasmic reticulum tubular network organization, synaptic vesicle clustering, and so forth. GO cellular component analysis indicated that most of the target genes were clustered into ESC/E(Z) complex, cyclin-dependent protein kinase holoenzyme complex, and pre-autophagosomal structure membrane. Analysis of GO molecular function category illustrated that a large proportion of the genes were involved in GPI-linked ephrin receptor activity, MAP kinase kinase kinase activity, nuclear localization sequence binding, and so on. In addition, the KEGG pathway analysis revealed that the target genes were enriched in various cancer-related pathways, such as mTOR signaling pathway, Wnt signaling pathway, cell cycle, p53 signaling pathway, TGF-beta signaling pathway, and so forth (Figure 3C). It is worth noting that the target genes of these miRNAs were also associated with non-small cell lung cancer and small cell lung cancer, suggesting that the chronic arsenite exposure confers some characteristics of lung cancer cells to normal human bronchial epithelial cells. 

### 3.5. The miRNA-Gene Regulatory Network

**Figure 4:**
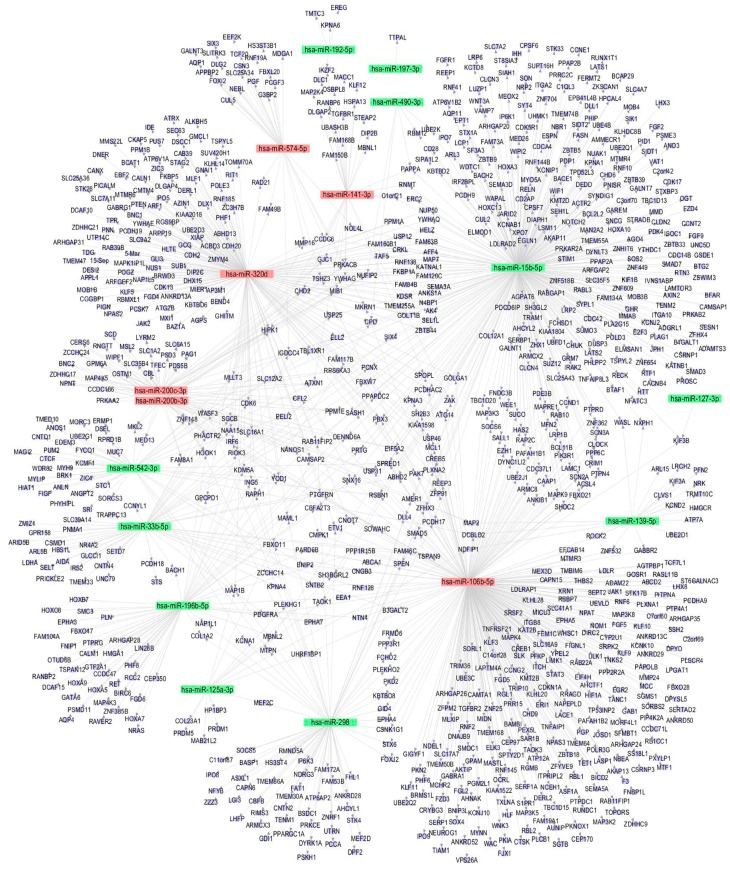
miRNA-gene network. Eleven downregulated and six upregulated miRNAs and their target genes were used to construct the regulatory network. Blue circle represents a gene (mRNA), red square represents an upregulated miRNA, and green square represents a downregulated miRNA. The relationship between miRNAs and genes is represented by one gray line.

The miRNA-gene regulatory network was constructed to show the interactions of the miRNAs and their targets via cytoscape software. As indicated in Figure 4, the target genes were mainly regulated by miR-15b-5p (338 genes), miR-106b-5p (316 genes), and miR-320d (177 genes), and these three miRNAs were the key node in the regulatory network. On the contrary, miRNAs like miR-197-3p, miR-127-3p, and miR-490-3p regulate few genes and are on the edge of the network, suggesting these miRNAs were less important in arsenite-transformed cells.

### 3.6. The Cell Cycle Distribution

**Figure 5:**
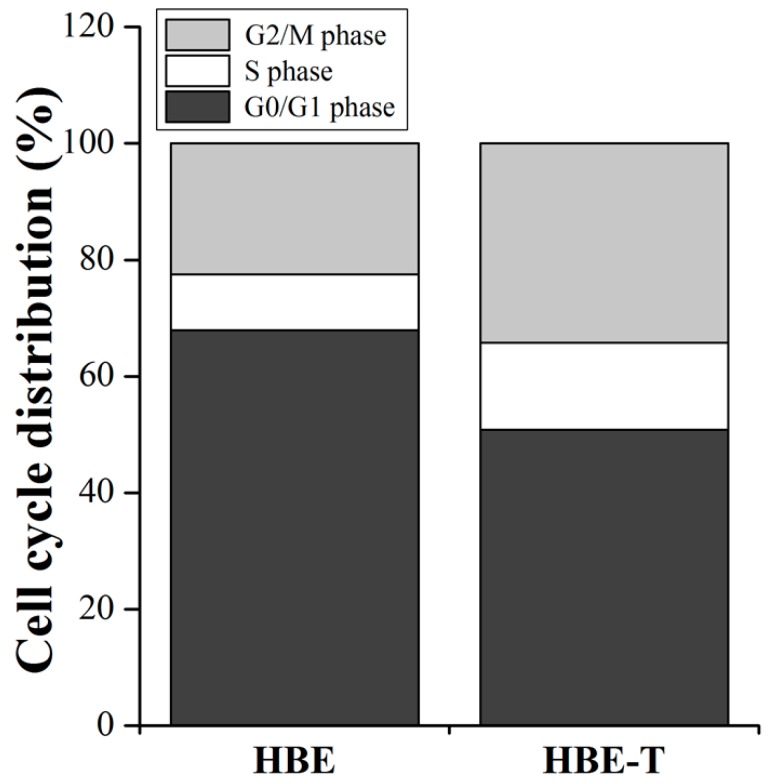
Cell cycle distribution in HBE-T and HBE cells. Cell cycle distribution in HBE and HBE-T cells was detected by flow cytometry using propidium (PI) staining. All the data were from three independent experiments and presented as mean ± standard deviation (SD). *p*-Values were calculated using independent samples *t* test. The difference of cell cycle distribution between HBE and HBE-T cells is statistically significant with *p* < 0.05.

Since the pathway enrichment analysis (Figure 3C) demonstrated that the cell cycle may regulate by differentially miRNAs, we further detected the cell cycle distribution in HBE and HBE-T cells to validate the prediction result. As shown in Figure 5, the percentage of HBE-T cells in G0/G1 phase was decreased when compared with that of HBE cells, while the percentage of HBE-T cells in S phase and G2/M phase were higher than those in HBE cells, suggesting that the cell cycle is indeed being regulated by these differentially miRNAs in arsenite-transformed cells. 

## 4. Discussion

Apoptotic inhibition, aberrant DNA repair and methylation, histone acetylation abnormity, excessive generation of reactive oxygen species, and alterations of signal transduction pathways have been proposed to be associated with arsenite-induced carcinogenesis [6,7,8,9,10,11]. About 50% of human miRNA genes are located at fragile sites and genomic regions associated with cancers, and this location implicates an important role of miRNAs in tumorigenesis [34]. Although earlier studies have demonstrated that some miRNAs were related to arsenite-induced carcinogenesis [3,4,10,18,19,20,21], the relationship between miRNAs and neoplastic transformation is still elusive. Global expression profile analysis of miRNAs may provide a unique opportunity to understand the potential miRNA regulatory mechanisms underlying carcinogenicity of arsenite. In this study, miRNA Array was used to detect the altered expression profile of miRNAs between parent and arsenite-transformed cells. Our results showed that a total of 191 aberrant miRNAs (2-fold changing) were identified in arsenite-transformed cells. This is consistent with the notion that altered miRNA expression was involved in arsenite-induced carcinogenesis [3,4,10,18,19,20,21]. In our results, two-thirds (128 miRNAs) of these aberrant miRNAs were downregulated, and the rest (63 miRNAs) were upregulated. This result is different from other study, in which a total of 30 miRNAs were significantly upregulated, whereas 21 miRNAs were markedly downregulated in arsenite-transformed cells (human bronchial epithelial cells, exposed to 1 μM sodium arsenite for 30 passages and 15 weeks) [21]. This suggests that different time and concentration of arsenite treatments were possibly responsible for this distinct miRNA expression pattern. More importantly, the change of miRNA expression profile in neoplastic transformation of human bronchial epithelial cells induced by arsenite was also different from other transformed cell types induced by arsenite [10] or the cells exposed to arsenite for a short time [35], despite some miRNAs that are not commonly dysregulated or dysregulated in opposite direction. These studies together demonstrated that the change of miRNA expression response to arsenite is potentially cell-type specific and the different time and concentration of arsenite treatments may contribute to distinct miRNA signature. Furthermore, the miRNA profile of the arsenite-induced neoplastic transformation in our study showed that a series of miRNAs is often dysregulated in lung cancer, e.g., let-7 family, miR-200 family, miR-125a, miR-145, miR-192, miR-145, miR-335 [36]. This fact further suggests that the miRNA spectrum of arsenite-transformed cells is similar with that of lung cancer cells. Althought the expression level of some miRNAs (e.g., miR-21 and miR-191) have been reported to regulate the neoplastic transformation induced by arsenite [18,21], their changes were less than 2-fold in our present study. This difference can be explained by the presence of certain variability in detection, and miRNAs that associated with arsenite carcinogenicity may have exposure concentration and cell type specificity. The expression levels and regulation mechanisms of these cancer associated miRNAs in arsenite-induced carcinogenesis should be further validated and explored. 

Until now, over 2000 miRNAs have been discovered, and their regulation on genes and signaling pathways are extremely complex [15,16,17]. Although studies have demonstrated that some miRNAs were involved in arsenite-induced neoplastic transformation by regulation oncogenes and/or tumor suppressor genes [3,4,10,18,19,20,21], the functions of miRNA target genes need to be analyzed comprehensively. Therefore, in this study, seventeen of differentially expressed miRNAs were selected to further examine the regulatory role of miRNAs in carcinogenicity of arsenite. The corresponding target genes of these miRNAs were predicted with the TargetMiner, miRDB, and TarBase databases. A total of 954 target genes were sorted from the interaction of these three databases. In order to understand the association of these dysregulated miRNA-gene pairs with the carcinogenicity of arsenite, KEGG pathway analysis was performed to integrate individual component into a unified pathway in this study. The analysis results showed that the 954 genes were enriched in tumor-associated pathways, such as non-small cell lung cancer, small cell lung cancer, thyroid cancer, pancreatic cancer, chronic myeloid leukemia, and so forth, suggesting that chronic arsenite exposure confers characteristics of tumor cells to HBE cells via regulating various cancer pathways. The results were supported by a previous report in which the cancer-associated pathways were also activated in the malignant transformed cells induced by cadmium [37]. 

Our results also demonstrated that signaling pathways which were closely related to proliferation, invasion, and metastasis of tumor cells were also modulated by miRNAs in arsenite-transformed cells. These signaling pathways included p53 signaling pathway, Wnt signaling pathway, cell cycle, mTOR signaling pathway, TGF-beta signaling pathway, and so forth. Consistently, previous study has been reported that p53 signaling pathway was involved in transformation induced by chronic arsenite exposure [8]. In addition, Wnt signaling pathway and TGF-beta signaling pathway have been evidenced to regulate the metastasis and invasion of tumor cells [38,39]. The activation of these two pathways by aberrant miRNAs may contribute to the elevated migration distance of arsenite-transformed cells. Previous research tends to support our predictions. 

To further confirm whether the predictions are reliable or not, the cell cycle distribution in parent and arsenite-transformed cells was detected by flow cytometry. As expected, the percentages of HBE-T cells in S phase and G2/M phase were higher than those in HBE cells (Figure 5). These results further confirmed the predictions of KEGG pathway analysis in which the aberrant miRNAs were involved in arsenite-induced neoplastic transformation by regulating the cell cycle. Results of GO analysis illustrated that the target genes of miRNAs were enriched in the three categories of biological process, cellular component, and molecular function. In line with the notion that autophagy has a significant contribution to malignant transformation [9,40], the GO categories enrichment results showed that the positive regulation of macroautophagy and pre-autophagosomal structure membrane, one of the biological process and cellular component, respectively, were related to the transformation induced by arsenite. This is also in accordance with the result from KEGG pathway analysis that autophagy may modulate the carcinogenesis of arsenite via the mTOR signaling pathway activation. In a word, based on KEGG pathway and GO analysis, the activation of signaling pathways regulated by miRNAs may contribute to formation and maintenance the neoplastic phenotype of arsenite-transformed cells.

The miRNA-gene network illustrated that miR-15b-5p (338 regulated genes), as well as miR-106b-5p (316 regulated genes) and miR-320d (177 regulated genes), may play key roles in arsenite-induced carcinogenesis. Although no direct evidence has shown that the three miRNAs are associated with neoplastic transformation, some studies have demonstrated that these miRNAs are closely related to lung cancer occurrence and progression [24,25,26]. Specifically, the high expression of miR-15b in lung cancer tissues is closely related to the cellular metastasis and the resistance to cisplatin [24]. MiR-106b was one of the significant difference miRNAs between recurrent lung adenocarcinomas and non-recurrent lung adenocarcinomas [26]. Also, studies have shown over-expression of miR-106b promotes cell migration and metastasis in hepatocellular carcinoma by activating epithelial-mesenchymal transition process [41]. Thus, the regulation mechanisms of the three miRNAs in arsenite-induced transformation is worthy for further exploring. 

## 5. Conclusions

In summary, our study showed a series of aberrant miRNAs and their potential roles in arsenite-transformed cells. These results might provide some new and meaningful candidate miRNAs and target genes for further investigating the carcinogenic mechanism of arsenite. Although GO and KEGG pathway analysis highlighted the possible involvement of miRNA-dysregulated genes in arsenite-induced transformation, further study is needed to confirm the regulatory network of miRNAs and their target genes. In addition, since the modulation of the neoplastic transformation by miRNAs is complex, more experimental studies are required to elucidate these regulatory roles. It is worth noting that the aberrant miRNAs were identified from only one miRNA Array detection in our study, and this may lead to false-positive results. Having replicates would allow the variance of the samples to be taken into account. In addition, miRNAs with large variances may be incorrectly identified as alterations using the fold-change approach, which may lead to overestimate the roles of miRNAs in arsenite-induced transformation. Increasing the number of repetitions of miRNA Array in each group and performing bioinformatics analysis for entire differentially expressed miRNAs will definitely be helpful in increasing the accuracy of the results and may reveal miRNAs-mediated biologic events that were previously not known in arsenite-induced tumorigenesis. This is the focus of our future research work.

## Supplementary Materials

The following are available online at www.mdpi.com/2073-4425/8/10/254/s1. Table S1: Differentially expressed miRNAs in control (HBE cells) and test (HBE-T cells) group (XLSX). Table S2: miRNAs for validation and Bioinformatics analysis (XLSX). Table S3: Functional classification of the target genes by GO analysis (XLSX). Table S4: Pathway enrichment analysis of the target genes by KEGG pathway. (XLSX), Table S5: Data for miRNA-gene regulatory network (XLSX). Figure S1: Detection of altered miRNAs in HBE and HBE-T cells.
